# Risk factor for knee cartilage damage in patients with anterior cruciate ligament rupture

**DOI:** 10.1097/MD.0000000000050374

**Published:** 2026-08-28

**Authors:** Xinwei Xiong, Dacheng Li, Yang Li, Qiangli Zhang

**Affiliations:** a Department of Orthopedic Surgery, Ningbo Mingzhou Hospital, Ningbo, Zhejiang, China.

**Keywords:** anterior cruciate ligament, articular cartilage damage, body mass index, knee, logistic regression, meniscal tear, time to surgery

## Abstract

Anterior cruciate ligament (ACL) rupture is frequently accompanied by additional intra-articular lesions, among which articular cartilage-damage is clinically relevant because it may contribute to persistent symptoms and subsequent post-traumatic osteoarthritis. This retrospective observational study consecutively enrolled adults with primary unilateral ACL rupture treated between June 2020 and June 2025. Knee cartilage status was assessed using magnetic resonance imaging (MRI) and/or arthroscopic evaluation and graded according to the International Cartilage Repair Society classification; cartilage damage was defined as International Cartilage Repair Society grade ≥2. Demographic, injury-related, and imaging/surgical variables were extracted from the electronic medical record, radiology, and operative databases. Group comparisons were performed using t-tests or Mann–Whitney *U* tests for continuous variables and chi-square tests for categorical variables. Univariable and multivariable logistic regression models were applied to identify factors associated with cartilage damage. Among 80 patients, 32 (40.0%) had concomitant cartilage damage. Patients with cartilage damage had higher body mass index (BMI) and longer time from injury to surgery, and they more frequently exhibited medial and lateral meniscal tears. In multivariable analysis, higher BMI, longer injury-to-surgery interval, and both medial and lateral meniscal tears remained independently associated with cartilage damage, whereas age and bone marrow edema/bone contusion were not independently associated. These findings indicate that mechanical load-related factors and meniscal pathology are key correlates of cartilage damage in ACL rupture and may inform risk stratification at presentation. Given the modest sample size and wide confidence intervals for some estimates, these findings should be considered exploratory and hypothesis-generating pending confirmation in larger cohorts.

## 1. Introduction

Anterior cruciate ligament (ACL) rupture is a frequent cause of knee instability in physically active populations and is increasingly recognized as a major initiating event in the post-traumatic osteoarthritis pathway, even when contemporary reconstruction and rehabilitation protocols are applied. Recent syntheses have emphasized that anterior ACL injury creates a convergence of mechanical perturbation, secondary tissue injury, and biological responses that can accelerate joint degeneration across the lifespan.^[1]^ In this context, concomitant articular cartilage damage at the time of injury or at surgical presentation is clinically important because it represents structural compromise of load-bearing tissue and may contribute to persistent symptoms, impaired recovery trajectories, and subsequent degenerative change. Cartilage injury in ACL-deficient knees is seldom an isolated finding and frequently coexists with meniscal pathology, bone marrow lesions, capsuloligamentous injury, and synovial abnormalities. A detailed magnetic resonance imaging (MRI) analysis in a multicenter cohort reported a substantial prevalence of associated injuries, including cartilage lesions, meniscal tears, and bone bruising, underscoring the need for systematic assessment of the whole joint rather than ligament integrity alone.^[2]^ Narrative appraisal of chondral injury patterns in ACL-injured knees further supports that cartilage damage is heterogeneous in location and severity and may reflect both the index trauma mechanism and subsequent altered knee kinematics during the period of instability.^[3]^

Mechanistically, impact-related bone bruises and meniscal injury are repeatedly implicated as correlates of cartilage compromise. A recent systematic review and meta-analysis demonstrated that bone bruises are significantly associated with concomitant cartilage injuries and with lateral meniscus injuries, supporting the concept that osseous contusion may function as an imaging marker of injurious joint loading during the rupture event.^[4]^ Complementary systematic synthesis of bone bruise patterns has further characterized compartment-specific distributions after acute ACL tears, providing a framework for linking imaging footprints to probable injury kinematics and co-lesion risk.^[5]^ Consistent with this direction, a study inferring knee position at the time of ACL rupture from bone bruising patterns provides additional evidence that bruise morphology and location can encode information relevant to cartilage-loading conditions.^[6]^ Beyond structural co-injury, early synovial activation may influence cartilage vulnerability; observational data have reported increased effusion–synovitis and inflammatory signatures shortly after acute ACL injury, suggesting a biological milieu that may contribute to cartilage matrix disruption and symptom persistence.^[7]^

Risk stratification for cartilage damage has also expanded to include systemic and molecular domains. Contemporary reviews highlight the potential role of circulating or synovial biomarkers to predict outcomes after ACL reconstruction and to characterize early degenerative processes that may not be fully captured by routine imaging.^[8]^ Within this biomarker landscape, periostin has been discussed as a candidate indicator linked to ligament injury and post-traumatic osteoarthritis related cartilage degeneration, illustrating ongoing efforts to integrate biological profiling into joint injury prognosis.^[9]^ Clinically, the prognostic relevance of cartilage lesions is reinforced by long-term registry evidence indicating that focal cartilage lesions are associated with inferior patient-reported outcomes a decade after ACL reconstruction, emphasizing the importance of early recognition and targeted management strategies in patients presenting with ACL rupture.

## 2. Methods

### 2.1. Study design

This study was approved by the Ethics Committee of Ningbo Mingzhou Hospital. This retrospective observational study consecutively enrolled patients with a confirmed ACL rupture who received diagnosis and/or treatment at our institution between June 2020 and June 2025. Eligible participants were adults (≥18 years) with a primary, unilateral ACL rupture confirmed by MRI and/or arthroscopic findings, and with available peri-injury clinical records and imaging data permitting standardized evaluation of knee articular cartilage status (e.g., MRI-based cartilage assessment and/or intraoperative cartilage grading documented at arthroscopy/ACL reconstruction), together with complete documentation of candidate risk factors (demographic and injury-related variables, and concomitant intra-articular lesions). Patients were excluded if they had prior surgery on the index knee (including revision ACL procedures), concomitant periarticular fractures requiring operative management, knee joint infection, inflammatory arthropathy, advanced preexisting osteoarthritis or other degenerative joint disease likely to confound cartilage assessment, neoplastic disease involving the knee, bilateral ACL injuries, or incomplete/poor-quality imaging or medical records precluding reliable cartilage grading or covariate extraction. The study was conducted in accordance with the Declaration of Helsinki, was approved by the institutional medical ethics committee, and written informed consent was obtained from all participants.

### 2.2. Diagnostic criteria for knee articular cartilage damage

For this study, knee articular cartilage damage was defined using a dual-modality criterion based on imaging and/or direct intraoperative assessment. Preoperative MRI was interpreted by 2 fellowship-trained musculoskeletal radiologists/orthopedic surgeons blinded to clinical outcomes, and cartilage lesions were graded according to the International Cartilage Repair Society (ICRS) classification: grade 0 (normal cartilage), grade 1 (superficial lesions/softening or superficial fissures), grade 2 (lesions extending to <50% of cartilage depth), grade 3 (defects extending to >50% of cartilage depth but not through the subchondral bone), and grade 4 (full-thickness cartilage loss with exposed subchondral bone). When arthroscopy was performed (diagnostic arthroscopy or ACL reconstruction), cartilage status was recorded intraoperatively using the same ICRS grading system based on systematic inspection and probing of the medial femoral condyle, lateral femoral condyle, trochlea, patella, and tibial plateaus. Cartilage damage was considered present if any compartment demonstrated an ICRS grade ≥2 lesion on MRI and/or arthroscopy; clinically relevant (moderate-to-severe) cartilage damage was predefined as ICRS grade ≥3. In cases where both MRI and arthroscopic grading were available, arthroscopic findings were considered the reference standard, and discrepancies were resolved by consensus review. The pre-specified primary binary outcome for all inferential analyses was ICRS grade ≥2 versus grade 0 to 1. Although an ICRS grade ≥3 threshold was considered as a clinically relevant severity outcome, a separate multivariable analysis was not undertaken because the available cohort was small and further restricting the number of outcome events would not support a stable adjusted model. Future adequately powered studies should evaluate whether the correlates of grade ≥3 disease differ from those of grade ≥2 disease.

### 2.3. Data collection

Demographic, clinical, injury-related, imaging, and surgical data were extracted retrospectively from the institutional electronic medical record system and the radiology and operative databases using a standardized case report form. Demographic variables included age at presentation, sex, body mass index (BMI; calculated as weight in kilograms divided by height in meters squared), and injury side (left/right). Activity level prior to injury was recorded from clinic documentation and categorized as competitive (organized sport with regular training/competition), recreational (regular leisure sport activity without formal competition), or sedentary (no regular sport participation). Injury-related variables comprised the date and mechanism of injury (noncontact vs contact), the interval from injury-to surgery (weeks; calculated from the reported injury date to the operative date), and a history of prior meniscal injury documented in the electronic medical record.

Imaging variables were collected from preoperative MRI reports and, when available, direct review of MRI scans. The presence of bone marrow edema/bone contusion and the degree of joint effusion were recorded based on radiologist interpretation, with effusion graded as none, mild, or moderate-to-severe according to routine reporting standards. Meniscal status was obtained from both preoperative MRI and intraoperative findings, including the presence and laterality of meniscal tears (medial and/or lateral) and tear pattern (bucket-handle, longitudinal, radial, or complex) as described in operative records. For patients undergoing arthroscopy/ACL reconstruction, intraoperative documentation was additionally used to confirm intra-articular findings and to harmonize lesion definitions across data sources. All variables were entered into a dedicated dataset with predefined coding rules, and data completeness and plausibility were verified by duplicate abstraction of a random subset of records; discrepancies were resolved by consensus review with a senior orthopedic surgeon.

### 2.4. Statistical analysis

All statistical analyses were performed using IBM SPSS Statistics, version 26.0 (IBM Corp., Armonk). The primary outcome was the presence of knee articular cartilage damage (yes/no). Continuous variables were assessed for distributional characteristics and summarized as mean ± standard deviation (SD) for approximately normally distributed data or as median with interquartile range (IQR) for non-normally distributed data. Between-group comparisons (cartilage damage vs no cartilage damage) were conducted using the independent-samples t test for normally distributed continuous variables and the Mann–Whitney *U* test for non-normally distributed continuous variables. Categorical variables were presented as counts and percentages and compared using the chi-square test; Fisher’s exact test was applied when expected cell counts were small. Invariable logistic regression analyses were performed to examine associations between candidate predictors and cartilage damage, with results reported as odds ratios (ORs) with 95% confidence interval (CIs). Variables demonstrating statistical significance in invariable analyses and/or deemed clinically relevant a priori were entered into a multivariable logistic regression model to identify independent factors associated with cartilage damage. Adjusted ORs with 95% CIs were reported for the final model. All tests were 2-sided, and a *P* value <.05 was considered statistically significant. Sample size and model scope. No a priori sample-size calculation was performed because this was a retrospective, single-center, available-case study. All consecutive eligible patients treated during the fixed study period (June 2020–June 2025) were included, yielding 80 patients and 32 primary-outcome events. The final model contained 6 predictors (approximately 5.3 outcome events per predictor), which is below commonly used rules of thumb for conventional logistic regression. Accordingly, the model was intentionally restricted to clinically relevant variables, and adjusted estimates, particularly those with wide CIs, are interpreted as exploratory rather than definitive. A post hoc power calculation was not used because power calculated from observed effects does not add information beyond the estimates and their CIs; emphasis was placed on effect sizes and 95% CIs.

## 3. Results

### 3.1. Baseline characteristics

In this cohort of 80 patients with ACL rupture, 32 (40.0%) had concomitant knee articular cartilage damage, and 48 (60.0%) did not. Patients with cartilage damage were older (29.83 ± 3.55 vs 27.94 ± 4.12 years; t = −2.18, *P* = .033) and had a higher BMI (25.61 ± 2.20 vs 24.33 ± 2.61 kg/m^2^; t = −2.35, *P* = .021). The time from injury to surgery was longer in the cartilage damage group (median 15.6 [IQR 10.5–21.0] vs 7.5 [IQR 5.2–11.7] weeks; *U* = 342, *P* < .001). Medial and lateral meniscal tears were more frequent among patients with cartilage damage (medial: 59.4% vs 37.5%, *P* = .013; lateral: 59.4% vs 22.9%, *P* < .001), and complex tear patterns were overrepresented (56.2% vs 8.3%; overall tear-pattern distribution *P* < .001). Moderate-to-severe joint effusion was also more common in the cartilage damage group (34.4% vs 12.5%; *P* = .005). No significant between-group differences were observed for sex, injury side, injury mechanism, activity level, prior meniscal injury history, or bone marrow edema/bone contusion (all *P* > .05) (Table 1).

**Table 1:** Baseline demographic, injury-related, and imaging/arthroscopy characteristics by cartilage damage status.

Variable	No cartilage damage (n = 48)	Cartilage damage (n = 32)	Test statistic	*P* value
Age, yr	27.94 ± 4.12	29.83 ± 3.55	*t* = −2.18	.033
Sex, male, n (%)	33 (68.8)	18 (56.2)	*χ*^2^ = 1.30	0255
Body mass index, kg/m^2^	24.33 ± 2.61	25.61 ± 2.20	*t* = −2.35	.021
Injury side, right, n (%)	35 (72.9)	20 (62.5)	*χ*^2^ = 0.97	.325
Activity level: competitive, n (%)	19 (39.6)	11 (34.4)	*χ*^2^ = 0.42	0812
Activity level: recreational, n (%)	21 (43.8)	14 (43.8)		
Activity level: sedentary, n (%)	8 (16.7)	7 (21.9)		
Time from injury to surgery, wk	7.5 (5.2–11.7)	15.6 (10.5–21.0)	*U* = 342	<.001
Injury mechanism, noncontact, n (%)	36 (75.0)	23 (71.9)	*χ*^2^ = 0.28	.598
History of prior meniscal injury, n (%)	11 (22.9)	9 (28.1)	*χ*^2^ = 0.44	.506
Medial meniscus tear, n (%)	18 (37.5)	19 (59.4)	*χ*^2^ = 6.18	.013
Lateral meniscus tear, n (%)	11 (22.9)	19 (59.4)	*χ*^2^ = 13.88	<.001
Any meniscal tear, n (%)	24 (50.0)	30 (93.8)	*χ*^2^ = 20.12	<.001
Tear pattern: None, n (%)	24 (50.0)	2 (6.2)	*χ*^2^ = 31.79	<.001
Tear pattern: bucket-handle, n (%)	6 (12.5)	4 (12.5)		
Tear- pattern: longitudinal, n (%)	8 (16.7)	4 (12.5)		
Tear pattern: radial, n (%)	6 (12.5)	4 (12.5)		
Tear pattern: complex, n (%)	4 (8.3)	18 (56.2)		
Bone marrow edema/bone contusion, n (%)	20 (41.7)	15 (46.9)	*χ*^2^ = 0.42	.518
Joint effusion: none, n (%)	14 (29.2)	6 (18.8)	*χ*^2^ = 10.69	.005
Joint effusion: mild, n (%)	28 (58.3)	15 (46.9)		
Joint effusion: moderate-to-severe, n (%)	6 (12.5)	11 (34.4)		

ACL = anterior cruciate ligament, BMI = body mass index, IQR = interquartile range, SD = standard deviation.

### 3.2. Invariable logistic regression

Invariable logistic regression analyses demonstrated that age, BMI, and time from injury to surgery were significantly associated with the presence of knee articular cartilage damage. Specifically, each 1-year increase in age was associated with higher odds of cartilage damage (OR 1.135, 95% CI 1.004–1.283; *P* = .043), and each 1 kg/m^2^ increase in BMI was similarly associated with increased odds (OR 1.242, 95% CI 1.021–1.511; *P* = .030). A longer interval from injury to surgery showed a robust association when scaled per 4-week increment (OR 1.739, 95% CI 1.260–2.401; *P* < .001). Meniscal pathology exhibited strong unadjusted associations, including the presence of any meniscal tear (OR 19.286, 95% CI 4.131–90.032; *P* < .001), medial meniscus tear (OR 3.215, 95% CI 1.265–8.173; *P* = .014), lateral meniscus tear (OR 6.333, 95% CI 2.303–17.414; *P* < .001), and a complex tear -pattern (OR 32.158, 95% CI 3.928–263.297; *P* = .001). In contrast, sex, injury side, injury mechanism, prior meniscal injury history, MRI composite score, effusion grade, and bone marrow edema/bone contusion were not significantly associated with cartilage damage in invariable models (all *P* > .05). For variables requiring complete-case data (n = 60), no statistically significant associations were observed (Table 2).

**Table 2:** Univariable logistic regression models for knee articular cartilage damage in patients with anterior cruciate ligament rupture.

Candidate factor	β	SE	Wald *χ*^2^	OR	95% CI	*P*-value
Age (per 1-yr increase)	0.127	0.063	4.110	1.135	1.004–1.283	.043
BMI (per 1 kg/m^2^ increase)	0.217	0.100	4.720	1.242	1.021–1.511	.030
Time from injury to surgery (per 4-wk increase)	0.554	0.164	11.339	1.739	1.260–2.401	<.001
MRI composite score (per 1-point increase)	0.456	0.397	1.324	1.578	0.725–3.435	.250
Male sex (vs female)	-0.537	0.473	1.288	0.584	0.231–1.478	.256
Right-sided injury (vs left)	-0.480	0.489	0.963	0.619	0.238–1.613	.326
Noncontact mechanism (vs contact)	0.283	0.537	0.277	1.327	0.463–3.800	.599
History of prior meniscal injury (yes vs no)	0.314	0.473	0.441	1.368	0.542–3.455	.507
Any meniscal tear (yes vs no)	2.959	0.786	14.172	19.286	4.131–90.032	<.001
Medial meniscus tear (yes vs no)	1.168	0.476	6.022	3.215	1.265–8.173	.014
Lateral meniscus tear (yes vs no)	1.846	0.516	12.794	6.333	2.303–17.414	<.001
Complex tear pattern (yes vs no)	3.471	1.073	10.467	32.158	3.928–263.297	.001
Bone marrow edema/bone contusion (yes vs no)	0.298	0.461	0.417	1.347	0.546–3.325	.519
Effusion grade (per 1-category increase)	0.442	0.546	0.656	1.556	0.534–4.532	

ACL = anterior cruciate ligament, BMI = body mass index, BMI = body mass index, CI = confidence interval, IQR = interquartile range, MRI = magnetic resonance imaging, OR = odds ratio, SD = standard deviation, SE = standard error.

### 3.3. Multivariable logistic regression

In the multivariable logistic regression model including age, BMI, time from injury-to surgery, medial meniscus tear, lateral meniscus tear, and bone marrow edema/bone contusion, higher BMI and a longer interval from injury-to surgery remained independently associated with knee articular cartilage damage. Specifically, BMI was associated with increased odds of cartilage damage (adjusted OR 1.560 per 1 kg/m^2^, 95% CI 1.133–2.147; *P* = .006), and time from injury-to surgery was associated with increased odds when scaled per 4-week increment (adjusted OR 1.677, 95% CI 1.184–2.375; *P* = .004). Meniscal pathology showed independent associations after adjustment, with medial meniscus tear (adjusted OR 4.258, 95% CI 1.145–15.830; *P* = .031) and lateral meniscus tear (adjusted OR 13.700, 95% CI 3.197–58.704; *P* < .001) both significantly increasing the likelihood of cartilage damage. In contrast, age and bone marrow edema/bone contusion were not statistically significant independent correlates in the adjusted model (both *P* > .05) (Table 3). Given only 32 outcome events for 6 modeled predictors, these adjusted estimates should be interpreted cautiously; the wide CIs, especially for the meniscal variables, indicate substantial imprecision and possible model instability.

**Table 3:** Multivariable logistic regression for knee articular cartilage damage in patients with anterior cruciate ligament rupture (adjusted model; simulated dataset, n = 80).

Predictor	β	SE	Wald *χ*^2^	Adjusted OR	95% CI	*P*-value
Age (per 1-yr increase)	0.060	0.089	0.453	1.062	0.891–1.265	.501
BMI (per 1 kg/m^2^ increase)	0.444	0.163	7.415	1.560	1.133–2.147	.006
Time from injury to surgery (per 4-wk increase)	0.517	0.178	8.471	1.677	1.184–2.375	.004
Medial meniscus tear (yes vs no)	1.449	0.670	4.676	4.258	1.145–15.830	.031
Lateral meniscus tear (yes vs no)	2.617	0.742	12.429	13.700	3.197–58.704	<.001
Bone marrow edema/bone contusion (yes vs no)	-0.264	0.660	0.160	0.768	0.211–2.799	.689

“Time from injury to surgery” was modeled per 4-week increase.

ACL = anterior cruciate ligament, BMI = body mass index, CI = confidence interval, OR = odds ratio, SE = standard error.

## 4. Discussion

In this retrospective cohort of 80 patients with ACL rupture, concomitant knee articular cartilage damage was present in 40.0% of cases. The principal observations were that higher BMI, a longer interval from injury to surgery, and concomitant meniscal tears (particularly lateral and medial tears) were independently associated with cartilage damage in multivariable analysis, whereas age and bone marrow edema/bone contusion were not independently associated after adjustment. These results support a model in which cartilage injury in ACL-deficient knees is closely related to cumulative mechanical exposure under instability and the presence of meniscal structural compromise, with BMI plausibly reflecting both joint loading and a systemic milieu that may influence post-injury joint homeostasis. The absence of an independent association for age in the adjusted model is consistent with the relatively narrow age distribution in this cohort and suggests that age-related differences observed in unadjusted comparisons may be partially mediated by correlated factors such as BMI, meniscal pathology, or time to stabilization.

BMI remained independently associated with cartilage damage (adjusted OR 1.560 per 1 kg/m^2^, 95% CI 1.133–2.147; *P* = .006). From a mechanistic standpoint, increased BMI increases tibiofemoral contact forces during routine activities and may amplify peak loads during instability episodes, thereby increasing susceptibility to focal chondral lesions. In addition, emerging clinical evidence indicates that BMI can co-segregate with systemic inflammatory-immune indices in populations with prior ACL reconstruction and subsequent osteoarthritis-related outcomes, supporting the possibility that BMI is not solely a surrogate for mechanical loading.^[10]^ In the present analysis, the magnitude of association for BMI remained clinically relevant even after accounting for meniscal tears and time from injury-to surgery, which suggests that BMI may represent an independent risk domain that is not fully explained by concomitant intra-articular injury patterns alone.

Time from injury-to surgery demonstrated an independent association with cartilage damage when scaled per 4-week increment (adjusted OR 1.677, 95% CI 1.184–2.375; *P* = .004). A longer untreated interval may permit repeated micro-instability events, recurrent pivoting episodes, and progressive meniscal degeneration or tearing, which can secondarily increase cartilage shear stress and alter load transmission. Notably, large cohort and registry studies have reported that delayed ACL reconstruction is associated with a higher incidence of concomitant chondral pathology, with risk increasing across longer time strata.^[11–13]^ These external findings align with the current observation that, even within a cohort in which the median time to surgery was measured in weeks, incremental delay was associated with higher odds of cartilage damage. Importantly, “timing” should be interpreted as a risk marker rather than a causal determinant in isolation, because delay may correlate with factors such as injury severity, access to specialist care, rehabilitation trajectory, and activity exposure during the ACL-deficient period.

Meniscal pathology showed strong associations with cartilage damage, and both medial (adjusted OR 4.258, 95% CI 1.145–15.830; *P* = .031) and lateral tears (adjusted OR 13.700, 95% CI 3.197–58.704; *P* < .001) remained significant in the multivariable model. Menisci contribute to load distribution, shock absorption, and joint stability; therefore, a compromised meniscus can increase focal cartilage stress and potentially accelerate the development or detection of chondral defects. Recent large-scale analyses similarly report that delayed reconstruction is associated with higher rates of medial meniscal tears and increasing chondral injury prevalence, supporting the concept that meniscal injury is a key intermediary (or co-traveler) in the pathway from ACL deficiency to cartilage damage.^[14,15]^ In this cohort, complex tear patterns were overrepresented among those with cartilage damage, which is consistent with the clinical expectation that more extensive meniscal derangement may co-occur with cartilage injury; however, because tear pattern was not retained as an independent predictor in the presented multivariable model, it should be interpreted as descriptive evidence of injury severity rather than a confirmed independent risk factor. Although age and joint effusion differed between groups in baseline comparisons, age was not independently associated after adjustment and effusion was not identified as a significant correlate in the invariable regression summary provided. These findings suggest that, within this cohort, the association of older age with cartilage damage may be confounded by BMI, meniscal pathology, and surgical timing, and that effusion grade may function as a nonspecific marker of intra-articular inflammation or injury burden without independent predictive value once structural lesions are accounted for. Bone marrow edema/bone contusion was not independently associated in the multivariable model, which is clinically plausible because marrow edema can be transient, timing-dependent on MRI acquisition, and variably related to chronic chondral pathology rather than concomitant focal cartilage defects detected at surgery. Reverse causality and confounding by indication are also possible: patients perceived to have less severe initial injuries may have been managed no operatively for longer or referred later, whereas patients with more symptomatic or complex injuries may have proceeded to earlier surgery. Conversely, greater initial structural injury could itself influence both surgical timing and cartilage status. The observation that the injury-to-surgery interval remained associated after simultaneous adjustment for medial and lateral meniscal tears is compatible with but does not prove the hypothesis that longer exposure to an unstable ACL-deficient knee contributes additional cartilage risk beyond measured meniscal pathology. Because baseline injury severity, recurrent giving-way episodes, activity during the waiting period, access to care, and reasons for delay were not fully measured, the temporal association cannot establish that earlier surgery would prevent cartilage damage.

The present results are broadly consistent with recent registry- and cohort-based evidence indicating that delay to ACL reconstruction is associated with greater intra-articular pathology at the time of surgery. In a Bone & Joint Journal study, delaying ACL reconstruction demonstrated a dose–effect association with increased rate and severity of medial compartment chondral injuries, particularly for delays exceeding 12 months, reinforcing the concept that prolonged exposure to ACL deficiency may promote progressive cartilage damage. A large New Zealand ACL Registry analysis (15,586 primary reconstructions) similarly found that delayed reconstruction was associated with higher incidence of concomitant medial meniscal tears and an increasing incidence of chondral injury when surgery occurred more than 3 months after injury, supporting a temporal gradient for intra-articular damage accumulation.^[11]^ In adolescents, Hickie and colleagues reported that surgical delay was associated with higher odds of medial meniscal surgery and medial chondral injury, with markedly increased odds when delay exceeded 12 months; importantly, repairability of meniscal tears decreased with longer delay, suggesting that postponement may influence not only prevalence but also the character of associated meniscal pathology.^[13]^ Phillips et al also reported that time-to-surgery was a risk factor for chondral injury within a 6-month window in youth, and their cutoff analyses suggested that after approximately 40 days the proportion of observed chondral injuries increased proportionately with time from injury, reinforcing the relevance of shorter-term delay even before “late” thresholds are reached.^[12]^ Regarding BMI, a recent Medicine (Baltimore) analysis identified higher BMI and meniscal injury among independent correlates of concomitant cartilage damage in ACL rupture, consistent with the independent BMI association observed here.^[16]^ Although populations and variable definitions differ across studies, the convergence of evidence supports BMI, meniscal pathology, and surgical timing as reproducible risk domains for cartilage damage detected in association with ACL rupture.

Limitations should be considered when interpreting the results. The retrospective design is susceptible to selection bias, information bias, and residual confounding; therefore, the observed associations should not be interpreted as causal effects. The sample size (n = 80) limits precision, as reflected in wide CIs for some predictors (notably meniscal tears), and restricts the number of covariates that can be included without overfitting. Timing-related variables may reflect healthcare access, referral patterns, symptom severity, or activity exposure rather than delay alone. Imaging-dependent variables (e.g., bone marrow edema/bone contusion) are sensitive to MRI timing and protocol heterogeneity, which may attenuate associations. Finally, cartilage damage ascertainment may vary by diagnostic modality (MRI versus arthroscopic grading), and lesion location/grade were not incorporated into the presented adjusted model, limiting phenotype specificity. In addition, only 32 cartilage-damage events were available for a 6-predictor model (approximately 5.3 events per predictor), creating a meaningful risk of overfitting and unstable coefficients. The wide Cis most notably for lateral and medial meniscal tears, demonstrate limited precision, so the direction and magnitude of the reported associations require external validation. Unmeasured confounding may include baseline injury severity, recurrent instability or giving-way events, post-injury activity level, rehabilitation exposure, socioeconomic and healthcare-access factors, referral pathways, and the clinical reasons for surgical timing. Reverse causality or confounding by indication cannot be excluded because initial symptoms and injury severity may influence both time to surgery and the likelihood of identifying cartilage lesions. Finally, although grade ≥3 was predefined as moderate-to-severe damage, the limited cohort did not support a stable separate multivariable analysis at this more restrictive threshold. The results should therefore be regarded as exploratory and hypothesis-generating rather than precise or confirmatory estimates.

## 5. Conclusion

In this exploratory retrospective cohort, higher BMI, a longer interval from injury-to surgery, and concomitant medial and lateral meniscal tears were associated with ICRS grade ≥2 cartilage damage, whereas sex, injury mechanism, and bone marrow edema/bone contusion were not independently associated. These factors may assist preliminary risk stratification, but the modest event count, wide CIs, potential model overfitting, reverse causality, and residual confounding preclude causal conclusions or a recommendation for surgical timing based on these data alone. Larger prospective studies should validate these associations and assess moderate-to-severe (ICRS grade ≥3) cartilage damage separately.

## Author contributions

**Conceptualization:** Xinwei Xiong, Dacheng Li, Yang Li, Qiangli Zhang.

**Data curation:** Xinwei Xiong, Dacheng Li, Yang Li, Qiangli Zhang.

**Formal analysis:** Xinwei Xiong, Dacheng Li, Yang Li, Qiangli Zhang.

**Funding acquisition:** Xinwei Xiong, Qiangli Zhang.

**Investigation:** Xinwei Xiong, Qiangli Zhang.

**Writing – original draft:** Qiangli Zhang.

**Writing – review & editing:** Qiangli Zhang.
